# The transcription factor ClWRKY61 interacts with ClLEA55 to enhance salt tolerance in watermelon

**DOI:** 10.1093/hr/uhae320

**Published:** 2024-11-11

**Authors:** Guangpu Lan, Changqing Xuan, Yidong Guo, Xin Huang, Mengjiao Feng, Li Yuan, Hao Li, Jianxiang Ma, Yong Zhang, Zhongyuan Wang, Jianqiang Yang, Rong Yu, Feishi Luan, Xian Zhang, Chunhua Wei

**Affiliations:** State Key Laboratory of Crop Stress Biology for Arid Areas, College of Horticulture, Northwest A&F University, Yangling 712100, Shaanxi, China; State Key Laboratory of Crop Stress Biology for Arid Areas, College of Horticulture, Northwest A&F University, Yangling 712100, Shaanxi, China; State Key Laboratory of Crop Stress Biology for Arid Areas, College of Horticulture, Northwest A&F University, Yangling 712100, Shaanxi, China; State Key Laboratory of Crop Stress Biology for Arid Areas, College of Horticulture, Northwest A&F University, Yangling 712100, Shaanxi, China; State Key Laboratory of Crop Stress Biology for Arid Areas, College of Horticulture, Northwest A&F University, Yangling 712100, Shaanxi, China; State Key Laboratory of Crop Stress Biology for Arid Areas, College of Horticulture, Northwest A&F University, Yangling 712100, Shaanxi, China; State Key Laboratory of Crop Stress Biology for Arid Areas, College of Horticulture, Northwest A&F University, Yangling 712100, Shaanxi, China; State Key Laboratory of Crop Stress Biology for Arid Areas, College of Horticulture, Northwest A&F University, Yangling 712100, Shaanxi, China; State Key Laboratory of Crop Stress Biology for Arid Areas, College of Horticulture, Northwest A&F University, Yangling 712100, Shaanxi, China; State Key Laboratory of Crop Stress Biology for Arid Areas, College of Horticulture, Northwest A&F University, Yangling 712100, Shaanxi, China; State Key Laboratory of Crop Stress Biology for Arid Areas, College of Horticulture, Northwest A&F University, Yangling 712100, Shaanxi, China; Institute of Horticultural Research, Ningxia Academy of Agriculture and Forestry Sciences, Yinchuan 750002, Ningxia, China; Key Laboratory of Biology and Genetic Improvement of Horticulture Crops (Northeast Region), Ministry of Agriculture and Rural Affairs, College of Horticulture and Landscape Architecture, Northeast Agricultural University, Harbin 150030, Heilongjiang, China; State Key Laboratory of Crop Stress Biology for Arid Areas, College of Horticulture, Northwest A&F University, Yangling 712100, Shaanxi, China; State Key Laboratory of Crop Stress Biology for Arid Areas, College of Horticulture, Northwest A&F University, Yangling 712100, Shaanxi, China

## Abstract

High salinity can severely inhibit the growth and development of watermelon (*Citrullus lanatus* L.). WRKY proteins are believed to mediate the adaptation of plants to abiotic stresses. Here, we identified the *ClWRKY61* gene, which positively regulates the tolerance of watermelon to salt stress. Knockout of the *ClWRKY61* reduced salt tolerance, while overexpression of the *ClWRKY61* enhanced salt tolerance in watermelon according to phenotypic and physiological analyses. Yeast two-hybrid assays revealed that ClWRKY61 interacts with the ClLEA55 protein, and this interaction was further confirmed by luciferase complementation imaging, transient bimolecular fluorescence complementation, and GST pull-down assays. Knockout of the *ClLEA55* resulted in lower salt tolerance compared to the wild-type plants. RNA-seq analysis indicated 421 up-regulated and 133 down-regulated genes in the *ClWRKY61* knockout line under salt stress, containing 293 differentially expressed genes with W-box in their promoters. Thirteen genes encoding phytoene synthase, MYB transcription factor, sucrose synthase, alpha/beta-hydrolases superfamily protein, glutathione reductase, sugar transporter, LEA protein, WRKY transcription factor, ERF transcription factor, alpha-glucan water dikinase, and calcium-dependent protein kinase showed transcriptional changes in *ClWRKY61* knockout line, *ClWRKY61* overexpression line, and *ClLEA55* knockout line under salt stress. These results provide an opportunity to mediate the regulation of salt stress in watermelon with WRKY proteins.

## Introduction

The salinization of arable land is a major problem in many countries. The global saline soil area approximately accounts for 8.7% of the world’s total area according to Food and Agriculture Organization statistics (2021). In China, the soil salinization area is estimated to comprise approximately 100 million hectares. The high content of salt in the soil adversely affects plants [1]. The extracellular osmotic pressure in high salt environments exceeds that within plant cells, hindering water absorption and causing physiological drought [2]. In addition, high ion concentrations enter cells through channels and carrier proteins, leading to ion toxicity [3]. For example, excessive Na^+^ inhibits the absorption of K^+^, which is essential for maintaining cell activity [4]. Furthermore, salinity causes decrease in photosynthesis, the accumulation of oxygen free radicals, and membrane destruction in plants [5–7].

Plants have evolved various mechanisms to withstand salinity and respond to other adverse environmental changes [8]. The Ca^2+^ signal is believed to act as a salt sensor in plants under salt stress [9]. The SALT OVERLY SENSITIVE (SOS) signaling is activated when cells sense salt-induced signal [10]. After sensing the cytosolic Ca^2+^ signal, Na^+^ is extruded by the SOS3 (CBL4)/SCaBP8 (CBL10)-SOS2 (CIPK24)-SOS1 component under salt stress [11]. In addition, the osmotic regulation mechanism mediates physiological changes in plants under salt stress [12]. The function of osmolytes is to maintain cell volume and pressure by osmoregulation, stabilize protein structures, act as oxygen free radicals scavengers, and protect cellular components [13]. These osmoprotectants include proline, quaternary ammonium compounds, sugars, and late embryogenesis abundant (LEA) protein [12–14].

The LEA protein family is the largest group of known intrinsically disordered proteins in plants [15]. LEA proteins have a flexible structure due to the absence of a tertiary structure. They are highly hydrophilic containing high levels of charged amino acid [16]. LEA proteins have been shown to mitigate the negative effects of abiotic stresses on plants [17–19]. LEA proteins are believed to protect cells from stress by chelating metal ions and reactive oxygen species (ROS), combining with the membrane, and safeguarding active enzymes via molecular shielding [20].

The WRKY transcription factor (TF) family, is predominantly identified in higher plants, participating in molecular regulation under abiotic stress [21]. WRKY TFs are generally characterized by the WRKYGQK motif and C_2_H_2_ or C_2_HC structure [22]. This conserved structure enables WRKY proteins to recognize and bind to the W-box, a specific DNA fragment represented as (C/T)TGAC(T/C) [23]. WRKY TFs are classified as subfamilies I, II, and III according to one or two WRKY structures and zinc finger motifs, with subfamily II further classified as IIa, IIb, IIc, IId, and IIe [24]. The expression of numerous WRKY family members, such as *AhWRKY75* in peanut (*Arachis hypogea* L.) [25], *GhWRKY17* in cotton (*Gossypium hirsutum* L.) [26], *TaWRKY75-A* in wheat (*Triticum aestivum* L.) [27], and *SlWRKY8* in tomato (*Solanum lycopersicum* L.) [28], is induced by salinity. This suggests underlying mechanisms by which WRKY proteins function under salt stress.

The function role of WRKY members under salt stress depends on a complex regulatory network. WRKY protein maintains Na^+^/K^+^ balance in cells by regulating the activity of ion homeostasis-related proteins such as SOS1, SOS2, SOS3, and high-affinity K^+^ transporter under salt stress [29–33]. WRKY proteins are also involved in the biosynthesis or signaling transduction pathways of various plant hormones in response to salt stress such as abscisic acid, auxin, and jasmonic acid [27, 34–36]. Additionally, the ROS scavenging system can be activated by WRKY TFs to enhance plant tolerance to salinity [25, 37, 38]. *MsWRKY33* improved the activity of oxidase to enhance salt tolerance in alfalfa (*Medicago sativa* L.). WRKY TFs are also central regulatory factors of the G protein signaling module involved in plant response to salt stress [39, 40]. Furthermore, RPD3-like histone deacetylase HDA9 and WRK53 are reciprocal negative regulators of each other’s activities, which regulate tolerance to salt stress in *Arabidopsis* [41].

Watermelon (*Citrullus lanatus* L.), valued for its significant economic impact, is widely cultivated and highly popular among consumers worldwide [42]. A total of 63 WRKY members have been identified in watermelon, classified into three groups (I–III) [43]. Here, we identified a gene, *ClWRKY61* (*Cla97C10G206240*), which is induced by salinity. Salt tolerance tests revealed that *ClWRKY61* acts as a positive regulatory factor. Furthermore, ClWRKY61 interacts with ClLEA55 and the *ClLEA55* knockout lines exhibit increased sensitivity to salt stress. In addition, we identified candidate genes that are potentially regulated by *ClWRKY61* through RNA-seq and qRT-PCR analysis. These findings enhance our comprehension of the molecular mechanisms by which WRKY proteins mediate the responses of watermelon to salt stress.

## Results

### The expression of *ClWRKY61* was induced by salinity in watermelon

**Figure 1:**
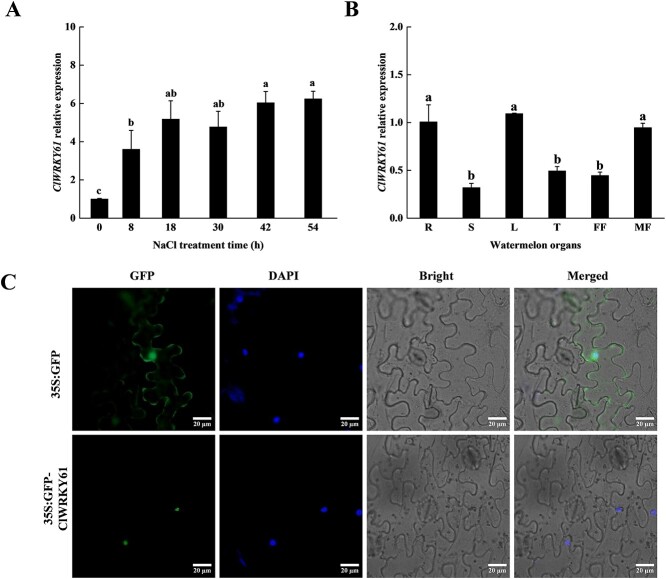
Expression and subcellular localization analysis of *ClWRKY61*. A) Expression analysis of *ClWRKY61* in watermelon under 300 mM NaCl treatments during a 54 h period. B) Expression analysis of *ClWRKY61* in organizations of watermelon. R is the root, S is the stem, L is the leaf, T is the tendril, FF is the female flower, and MF is the male flower. C) The GFP-ClWRKY61 protein was localized in the nucleus. DAPI is a nucleus indicator, and pGreenII-empty acts as a control. Statistical analysis by one-way ANOVA (*P* < 0.05)

*ClWRKY61*, an 831-bp full-length coding sequence (CDS) encoding 277 amino acids and a member of subfamily III, characterizes one WRKY structure and a C-X_7_-C-X_23_-H-X-C zinc-finger structure (Fig. S1A). Phylogenetic analysis revealed that it was most closely related to the *CsWRKY27* form cucumber (*Cucumis sativus* L.) (Fig. S1B), which could be dramatically induced by salt stress [44]. Moreover, *AtWRKY70*, as the orthologue of *ClWRKY61* (Fig. S1B), has been reported to play a role under salt stress in *Arabidopsis* [45]. To predict the potential function of *ClWRKY61*, we analyzed the expression pattern of *ClWRKY61* in various watermelon organs and explored its expression level under salt stress. Salt-induced experiments revealed that *ClWRKY61* expression was significantly increased after 8 h of stress (Fig. 1A), similar to the results in our previous research [43]. Furthermore, *ClWRKY61* was found to be significantly enriched in roots, leaves, and male flowers (Fig. 1B). Subcellular localization analysis showed that ClWRKY61 is localized to the nucleus of epidermal cells in tobacco (*Nicotiana benthamiana*) using a fluorescence microscope (Fig. 1C). These results suggested that the ClWRKY61 potentially plays a significant role in response to salt stress in watermelon.

### *ClWRKY61* positively regulates salt tolerance in watermelon

**Figure 2:**
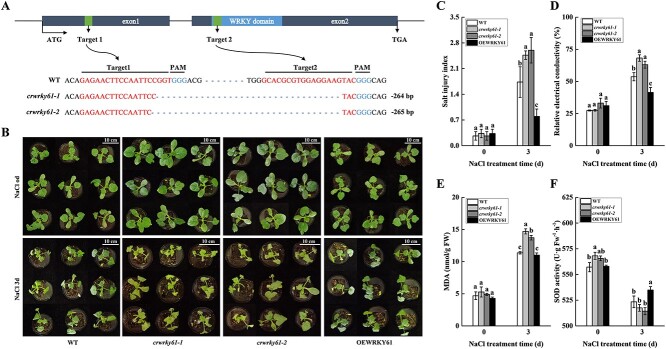
Phenotypic and physiological analysis of knockout and overexpression lines of *ClWRKY61* under 300 mM NaCl treatment at 3 days. A) Schematic representation of *ClWRKY61* with editing targets. *crwrky61-1* represents the 264-bp deletion, and *crwrky61-2* represents the 265-bp deletion. B) Phenotypic comparison of knockout, overexpression, and wild-type (WT) seedlings grown under 300 mM NaCl irrigation for 3 days. Scale bars, 10 cm. C) Salt injury index (SI) of knockout, overexpression, and WT seedlings grown under 300 mM NaCl irrigation for 3 days. D) Relative electrical conductivity comparison of knockout, overexpression, and WT seedlings grown under 300 mM NaCl irrigation for 3 days. E) MDA content of knockout, overexpression, and WT seedlings grown under 300 mM NaCl irrigation for 3 days. F) SOD activity of knockout, overexpression, and WT seedlings grown under 300 mM NaCl irrigation for 3 days. Statistical analysis by one-way ANOVA (*P* < 0.05)

*ClWRKY61* was found to contain two exons and one intron (Fig. 2A). To determine its biological function, we constructed knockout and overexpression vectors. Two *ClWRKY61* gene knockout lines, carrying homozygous alleles with a 264 and 265 bp deletion, were obtained using the CRISPR/Cas9 systems and genetic transformation methods (Fig. 2A). The *crwrky61-1* line had a deletion of 88 amino acids between the two target sites (Fig. S2A). The *crwrky61-2* line had a deletion of 265 bp between the two sgRNA sites, resulting in a premature translation termination and producing a protein with 47 amino acids (Fig. S2B). The overexpression vector contained the CDS of *ClWRKY61* driven by the *35S* promoter and GFP driven by another *35S* promoter (Fig. S1C). The overexpression lines were validated through GFP and qRT-PCR (Fig. S2D and E).

After being treated with 300 mM NaCl solution for three days, seedlings from both the *crwrky61-1* and *crwrky61-2* lines displayed more shriveling compared to wild-type (WT) plants (Figs 2B and S3), which was consistent with the trend of salt injury index (SI) analysis (Fig. 2C). However, the OEWRKY61 line outperformed WT plants (Figs 2B and S3), exhibiting the lowest SI at 3 days after treatment (Fig. 2C). We then characterized physiological changes under salt stress. Before salt treatment, there were no significant differences in relative electrical conductivity (REC) and malondialdehyde (MDA) content among the WT, *ClWRKY61* knockout lines, and *ClWRKY61* overexpression lines, whereas the superoxide dismutase (SOD) activity was elevated in the two knockout lines (Fig. 2D-F). Following three days of exposure to salt stress, compared to WT seedlings, both REC and MDA content markedly increased in the *crwrky61–1* and *crwrky61–2* lines but decreased in the OEWRKY61 lines (Fig. 2D, E). Furthermore, the activity of SOD was significantly higher in the OEWRKY61 lines but lower in the two gene-editing lines (Fig. 2F).

### ClWRKY61 interacts with ClLEA55 protein

**Figure 3:**
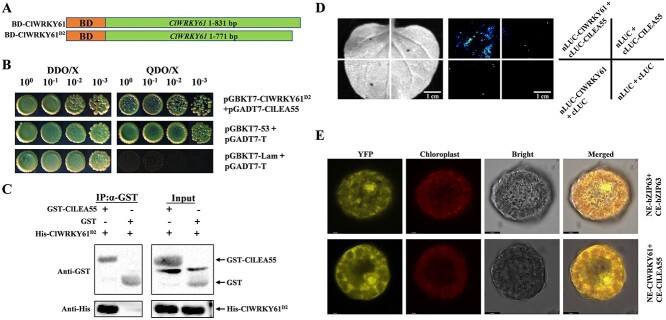
Interaction of ClWRKY61 with ClLEA55 at the protein level. A) Construction of pGBKT7 (BD) vector for *ClWRKY61*. *ClWRKY61^D2^* is *ClWRKY61* with 60 bp of the base from the C-terminus removed. B) Yeast-two-hybrid (Y2H) assay. ClWRKY61^D2^ interacted with ClLEA55 on selective media (DDO/X, SD/−Trp/−Leu/X-α-gal; QDO/X, SD-Ade/-His/−Leu/−Trp/X-α-gal). pGBKT7-p53 and pGADT7-RecT groups were positive controls. pGBKT7-Lam and pGADT7-RecT groups were negative controls. C) GST pull-down assay. GST-empty and GST-ClLEA55 proteins were incubated with His-ClWRKY61^D2^. Immunoblotting using anti-GST and anti-His antibodies. GST-empty + His-ClWRKY61^D2^ served as negative controls. D) Luciferase complementation imaging (LCI) assay. nLUC-ClWRKY61 and cLUC-ClLEA55 were coinjected into tobacco leaves to observe the luciferase signal. The remaining combinations served as negative controls. Scale bars, 1 cm. E) BiFC assay. NE-ClWRKY61 and CE-ClLEA55 were co-transformed into watermelon protoplasts, and fluorescence was observed. The NE-bZIP63 and CE-bZIP63 combination served as the positive control

To study whether the function of *ClWRKY61* depends on other proteins under salt stress in watermelon, we conducted a series of yeast-two-hybrid (Y2H) library assays with the ClWRKY61 protein as bait (BD-ClWRKY61) to identify potential interactions. We found that the C-terminus of *ClWRKY61* exhibited self-activation in SD/−Trp/X-α-Gal/200_ng/mL_ AbA medium (data not shown). The self-activation phenomenon was eliminated after removing 60 bp from the C-terminus of *ClWRKY61*, which was referred to as *ClWRKY61^D2^* (Fig. S4). Subsequently, we used ClWRKY61^D2^ as bait (BD-ClWRKY61^D2^) to identify 53 candidate proteins through Y2H assays (Table S1). Previous studies have shown that LEA proteins are induced to accumulate under salt stress [19, 46]. Hence, we tested the interaction of *ClLEA55* (*Cla97C09G168120*), which encodes late embryogenesis abundant protein, with ClWRKY61^D2^. The CDS sequence of *ClLEA55* was cloned into the prey vector (AD-ClLEA55). The BD-ClWRKY61^D2^ and AD-ClLEA55 vectors were co-transformed into Y2H strain and blue spots were observed on two deficiencies and four deficiencies selective media (Fig. 3B). We then performed GST pull-down assay. The recombinant His-ClWRKY61^D2^ protein was affined with the recombinant GST-ClLEA55 protein and the GST control. After proteins were eluted using GST-tag beads, antibodies were used to immunoblot the protein. The results showed that GST-ClLEA55 was pulled down, but not the GST control, indicating that His-ClWRKY61^D2^ interacted directly with the GST-ClLEA55 protein (Fig. 3C). The luciferase complementation imaging (LCI) experiment was used to further verify the interaction between nLUC-ClWRKY61 and cLUC-ClLEA55 in tobacco leaves, and the LUC luminescence signal was observed (Fig. 3D). The bimolecular fluorescence complementation (BiFC) assay showed a YFP fluorescent signal in the co-transformation of NE-ClWRKY61 and CE-ClLEA55 in watermelon protoplast, with NE-bZIP63 and CE-bZIP63 groups serving as positive controls (Fig. 3E). Overall, these findings revealed that ClWRKY61 and ClLEA55 formed a protein–protein complex *in vivo* and *in vitro*.

### *ClLEA55* positively regulates salt tolerance in watermelon

**Figure 4:**
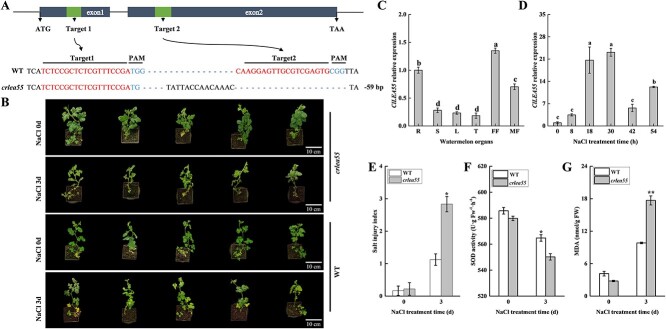
Expression analysis of *ClLEA55*, phenotypic and physiological analysis of *ClLEA55* knockout lines under 300 mM NaCl treatment at 3 days. A) Schematic representation of *ClLEA55* with editing targets. The sgRNA and the PAM sites are red and blue, respectively. *crlea55* represents the 59-bp deletion. B) Phenotypic comparison of *ClLEA55* knockout and WT seedlings grown under 300 mM NaCl irrigation for 3 days. Scale bars, 10 cm. C) Expression analysis of *ClLEA55* in watermelon organs. R is the root, S is the stem, L is the leaf, T is the tendril, FF is the female flower, and MF is the male flower. D) Expression analysis of *ClLEA55* in watermelon under 300 mM NaCl treatments during a 54 h period. E) Salt injury index of *ClLEA55* knockout and WT seedlings grown under 300 mM NaCl irrigation for 3 days. F) SOD activity of knockout and WT seedlings grown under 300 mM NaCl irrigation for 3 days. G) MDA content of ClLEA55 knockout and WT seedlings grown under 300 mM NaCl irrigation for 3 days. Statistical analysis by one-way ANOVA and Student’s *t*-test (*P < 0.05, **P < 0.01).

LEA proteins in watermelon were classified into seven groups (LEA 1–5, dehydrin, and SMP) and ClLEA55 is a subfamily III member containing a 41-amino acid motif [47]. We evaluated the expression of the *ClLEA55* in watermelon organs. The results indicated that *ClLEA55* exhibited high expression levels in roots, female flowers, and male flowers (Fig. 4C). In addition, *ClLEA55* expression was strongly up-regulated at 18 h after salt exposure (Fig. 4D). In the phylogenetic tree, *ClLEA55* was most closely related to the cucumber *CsLEA73* gene (Fig. S5A). To further validate the biological function of *ClLEA55* under salt stress in watermelon, a knockout line *crlea55* carrying a homozygous allele with a 73 bp deletion and a 14 bp insertion (Fig. 4A) was obtained with a truncated protein of 23 amino acids owing to a premature translation termination using CRISPR/Cas9 and genetic transformation methods (Fig. S6). Under normal growth conditions, no significant differences in phenotype and SI were observed between knockout and WT plants (Fig. 4B and E). After being subject to salt stress, the *crlea55* lines exhibited more shriveled appearance and significantly increased in the SI compared to WT seedlings (Fig. 4B and E). In addition, there was no significant difference between *ClLEA55* knockout and WT seedlings in MDA content and SOD activity under nonstress conditions (Fig. 4F and G). However, the *crlea55* seedlings displayed a significant increase in MDA content and a notable decrease in SOD activity compared to WT seedlings under NaCl stress (Fig. 4F and G). Compared to WT plants, *ClLEA55* expression was higher in the *ClWRKY61* overexpression lines and lower in the knockout lines according to qRT-PCR analysis (Fig. S5B). Similarly, *ClWRKY61* expression was lower in the *ClLEA55* knockout lines than in the WT plants (Fig. S5C). These findings indicate that the ClWRKY61-ClLEA55 complex may synergistically promote resistance to salt stress in watermelons.

### Multiple biological processes involved in *ClWRKY61*-induced salt stress resistance

**Figure 5:**
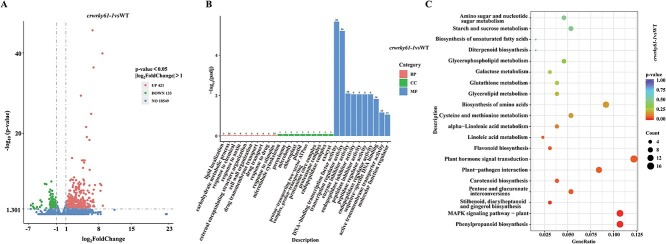
RNA-seq assays of *crwrky61-1* and WT lines under salt stress. A) DEGs volcano map. B) GO enrichment assays. Only the 30 terms most significantly enriched pathways are shown. The horizontal axis shows the GO term, and the vertical axis shows the significance level. BP, biological processes; CC, cellular components; MF, molecular functions. C) KEGG pathway assays. Only the 20 most significantly enriched pathways are shown. The horizontal axis shows the proportion of DEGs with the corresponding annotation, and the vertical axis shows the KEGG pathway. The size of the dots indicates the number of genes annotated to the KEGG pathway, and the color indicates the significance of enrichment

To identify genes potentially associated with salt tolerance, *crwrky61–1* and WT seedlings were exposed to salinity for 3 days and subjected to RNA-Seq analysis. Approximately 256 million clean reads totaling 38.34 Gbp were generated by Illumina sequencing (Table S2). A total of 554 differentially expressed genes (DEGs) (|log_2_FoldChange| ≥ 1.0, *P* ≤ 0.05) were identified, including 421 up-regulated and 133 down-regulated genes in the wild-type versus knockout line comparison group (Fig. 5A, Table S3).

We evaluated the functionality of DEGs through GO enrichment analysis. The results revealed that lipid localization, carbohydrate metabolic process, response to chemical, response to auxin, external encapsulating structure organization, cell wall organization, drug transport, response to drug, and response to stimulus were enriched in biological process. Microtubule cytoskeleton, peroxisome, microbody, chloroplast, plastid, proton-transporting domain, proteasome complex, and exocyst were enriched in cellular component. Transcription regulator activity, enzyme inhibitor activity, endopeptidase regulator activity, active transmembrane transporter activity, and molecular function regulator were enriched in molecular function (Fig. 5B). Functional assessment of the DEGs by KEGG pathway assays showed that DEGs mainly participated in phenylpropanoid biosynthesis, MAPK signaling pathway, stilbenoid, diarylheptanoid and gingerol biosynthesis, pentose and glucuronate interconversions, carotenoid biosynthesis, plant–pathogen interaction, and plant hormone signal transduction (Fig. 5C).

WRKY TFs can recognize the W-box element [23]. Among the DEGs, there were 221 up- and 72 down-regulated genes were identified with W-box sequence (Tables S4 and S5). To further discover the potential down-stream targets of *ClWRKY61*, we selected thirteen DEGs containing W-box *cis*-elements (Figs S7 and S8), including TFs (MYB, ERF, WRKY), synthase, kinase, reductase, hydrolase, LEA protein and transporter protein (Tables S6 and S7).

**Figure 6:**
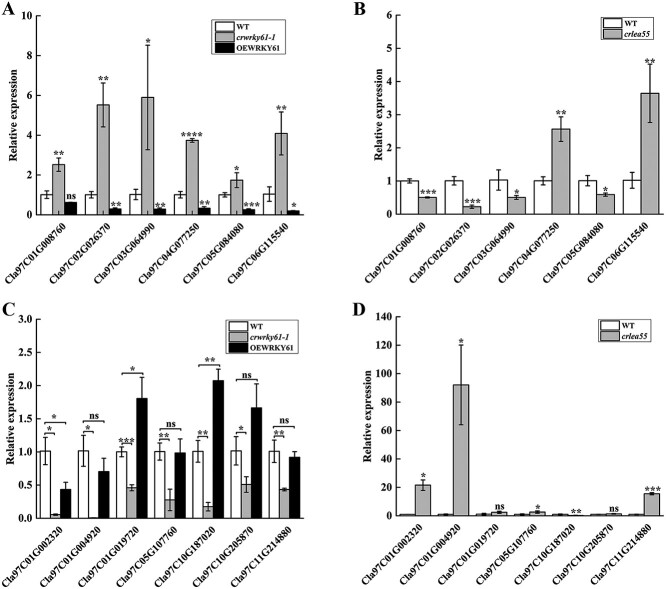
Expression verification of RNA data through qRT-PCR. A) The expression of some up-regulated DEGs in *ClWRKY61* transgenic lines. B) The expression of some up-regulated DEGs in *ClLEA55* knockout lines. C) The expression of some down-regulated DEGs in *ClWRKY61* transgenic lines. D) The expression of some down-regulated DEGs in *ClLEA55* knockout lines. Asterisks (Student’s *t*-test, *P < 0.05, **P < 0.01, ***P < 0.001, ****P < 0.0001) mean statistically significant differences, and ns represents no significance

These genes are recognized as playing a pivotal role in the responses of plants to salinity [46, 48–51]. Under salt stress, the transcriptional levels of *Cla97C01G008760* (phytoene synthase), *Cla97C02G026370* (MYB TF), *Cla97C03G064990* (sucrose synthase), *Cla97C04G077250* (alpha/beta-hydrolases superfamily protein), *Cla97C05G084080* (glutathione reductase), and *Cla97C06G115540* (sugar transporter) were significantly up-regulated in *crwrky61-1* lines but down-regulated in OEWRKY61 lines when compared to the WT (Fig. 6A). In the *crlea55* lines, the expression of *Cla97C01G008760*, *Cla97C02G026370*, *Cla97C03G064990*, and *Cla97C05G084080* were significantly lower while the expression of *Cla97C04G077250* and *Cla97C06G115540* significantly higher than the WT under NaCl treatment (Fig. 6B). Furthermore, Under NaCl treatment, the *crwrky61-1* lines exhibited significant down-regulation in the expression of *Cla97C01G002320* (LEA protein), *Cla97C01G004920* (ERF TF), *Cla97C01G019720* (protein kinase), *Cla97C05G107760* (water dikinase), *Cla97C10G187020* (ERF TF), *Cla97C10G205870* (WRKY TF), and *Cla97C11G214880* (ERF TF) compared to the WT (Fig. 6C). However, the expression of *Cla97C01G019720* and *Cla97C10G187020* in OEWRKY61 lines showed higher than the WT (Fig. 6C). In addition, compared to the WT, the expression of *Cla97C01G002320*, *Cla97C01G004920*, *Cla97C05G107760*, and *Cla97C11G214880* significantly increased while the expression of *Cla97C10G187020* significantly decreased in *crlea55* lines under salt stress (Fig. 6D).

## Discussion

As sessile organisms, watermelons are easily exposed to various types of stresses such as diseases, pests, drought, salinity, and cold. Consequently, they have evolved various mechanisms to adapt to these stresses. TFs are activated and perform function in regulating metabolism, growth, and development when plants are exposed to extreme environments [52]. WRKY proteins are mainly found in higher plants and participate in the adaptation and response to abiotic stress [53–55]. Here, we focused on *ClWRKY61*, which is a subfamily III WRKY TFs in watermelon. *AtWRKY70*, as the orthologue of *ClWRKY61*, responds to biotic stress through the salicylic acid pathway [56]. The experimental results revealed that *ClWRKY61* is significantly induced by salinity in watermelon and is localized to the nucleus (Fig. 1). Phenotypic and physiological analyses showed that the NaCl tolerance was lower in the two *ClWRKY61* mutations and higher in *ClWRKY61* overexpression lines than WT seedling (Fig. 2). Thus, *ClWRKY61* is a positive regulatory factor in the adaptation of watermelon to NaCl stress. Many WRKY family members have been found to be induced by salinity at the transcriptional level [27, 57, 58]. Under salt stress, WRKY proteins often interact with other proteins to achieve their functions. For example, *AtWRKY8* and *AtVQ9* have antagonist functions in mediating response to salt stress by ion homeostasis and gene regulation in *Arabidopsis* [59]. In maize (*Zea mays*), ZmWRKY20 and ZmWRKY115 proteins interact in the nucleus and repress *ZmbZIP111* transcription by binding to its promoter, which decreases the tolerance of plants to salinity [60]. In addition, WRKY TFs regulate the transcription of downstream genes in response to salinity stress. For instance, *AtWRKY46* positively mediates the development of the lateral roots by regulating ABA signal pathway and auxin homeostasis under salt stress [61]. *PalWRKY77* inhibits ABA response under salt stress in *Populus* [36]. ZmWRKY104 activates the transcription of superoxide dismutase gene *ZmSOD4* by targeting W-box and alleviates salt-induced cell damage in maize [62]. These studies indicate that the regulation of WRKY proteins to stress depends on a complex signaling and regulatory network.

We identified the candidate protein ClLEA55, which interacts with ClWRKY61, using a Y2H assay (Fig. 3B). This interaction was validated by both *in vivo* and *in vitro* assays (Fig. 3). The transcription level of the *ClLEA55* was enriched in watermelon roots and significantly induced by salinity, and its mutant watermelon seedlings showed lower tolerance to salt stress (Fig. 4). Plants encounter abiotic stress such as drought, salinity, and low temperature, which promote the secretion of LEA protein [63]. *AtLEA5* is the orthologue of *ClLEA55* in *Arabidopsis*, which reduces cellular oxidative damage caused by stress [64]. LEA proteins are intrinsically disordered, characterized by disorder and dynamism [15]. Due to their flexible structure, they participate in membrane protection, molecular shielding, and protein stability under stress [20]. LEA proteins are enriched in the early stages of salt stress in rice (*Oryza sativa* L.) and activated during the subsequent growth stages [46]. Recombinant *Escherichia coli* introduced into LEA protein display higher cell viability than control strains without the LEA protein in 5% and 7% NaCl medium [19]. Under salt stress, we speculate that the ClLEA55 protein maintains the normal function of the ClWRKY61 protein and cellular homeostasis. Therefore, *ClWRKY61* and *ClLEA55* may synergistically mediate the response to salinity stress in watermelon.

After that, we analyzed the transcriptional regulatory mechanism of *ClWRKY61* under salt stress by comparing *ClWRKY61* knockout and WT seedlings using RNA-Seq (Fig. 5). According to RNA-Seq analysis, *ClWRKY61* may respond to salt stress through biological processes such as biomolecular synthesis, substance transport, transcription regulation, carbohydrate metabolism, and phytohormone signal transduction. We examined the expression levels of genes that potentially serve as downstream targets of *ClWRKY61* in the *crwrky61–1*, OEWRKY61, and *crlea55* lines (Fig. 6). These genes encode TFs, phytoene synthase, sucrose synthase, hydrolase, glutathione reductase, sugar transporter, LEA proteins, and kinase. Metabolites such as sugars, glutathione, polyols, and amino acids serve in osmotic regulation, cell turgor pressure maintenance, signaling molecules, carbon storage, and ROS scavenging under salt stress [65, 66]. In addition, MYB, WRKY, and ERF TFs are generally considered to modulate plant adaptation to salt stress. These TFs mainly mediate gene response, hormone signal transduction, ion homeostasis, oxidation–reduction, and osmotic regulation under salt stress [49–51]. Calcium-dependent protein kinase contributes to the regulation of Na^+^ levels and antioxidant systems under salt stress [67]. We identified potential candidate genes for the downstream pathways regulated by *ClWRKY61* under salt stress.

In summary, the transcription levels of *ClWRKY61* and *ClLEA55* are induced by salinity stress. The synergistic functions of ClWRKY61 and ClLEA55 alleviate the damage to plant cells caused by salinity. RNA-seq and qRT-PCR assays indicate that *ClWRKY61* may mediate watermelon’s response to salinity stress via transcriptional regulation, signal transduction, substance transport, and metabolism.

## Materials and methods

### Plant materials and growth conditions

In this study, the watermelon inbred lines ‘YL’ and ‘TC’, as well as tobacco (*N. benthamiana*) were used as plant materials. First, watermelon seeds were soaked for 4 h and germinated under dark conditions at 28°C. Subsequently, the germinating watermelon seeds were transplanted into seedling pots and cultivated under 16-h light at 28°C and 8-h dark at 20°C photoperiod. Tobacco was cultivated under 16-h light and 8-h dark photoperiod at 25°C for approximately six weeks.

### Salt treatment and measurement of indicators

For salt treatment, watermelon seedlings at the three-leaf stage were irrigated with 300 mM NaCl solution, which has been widely utilized for salinity stress [7, 43, 68]. After the treatment, the second mature leaves were sampled with three biological replicates at 0, 8, 18, 30, 42, and 54 h, respectively. For the salt tolerance assays, seedlings of the *ClWRKY61* knockout and overexpression lines were treated with 300 mM NaCl solution during the three-leaf stages. For the *ClLEA55*, explant of *crlea55* and wild-type cultured in MS medium were transplanted into seedling pots after rooting. The seedlings were treated with 300 mM NaCl solution after the rejuvenation period. At 0 and 3 days, the second mature leaf was sampled with three biological replicates and stored at −80°C until further analysis. In addition, photos were taken and indicators such as REC, MDA content, SOD enzyme activity, and SI were measured by the previously described method with slight modification [69–71].

Watermelon leaves were completely soaked in 20 ml distilled water for 30 min. The conductivity of the solution was then determined and recorded. Next, the solution containing leaves was then boiled for 30 min, and the conductivity of the solution was determined and recorded again after cooling. The ratio of the conductivity before boiling to after boiling is the REC.

MDA content was measured using the following procedure. First, 0.2 g of watermelon leaves were ground in 5 ml phosphate buffer (50 mM, pH 7.8, 4°C). The homogenate was centrifuged at 12 000 rpm for 15 min at 4°C. Subsequently, 2 mL supernatant was mixed with 2 ml of 0.5% thiobarbituric acid and then boiled for 15 min. After cooling to room temperature, the samples were centrifuged for 15 min at 5000 g. The absorbance was determined and recorded at 532 and 600 nm.

SOD activity was measured using the following procedure. First, 0.2 g of watermelon leaves were ground in 5 m phosphate buffer (50 mM, pH 7.8, 4°C). The homogenate was centrifuged at 12 000 rpm for 15 min at 4°C. Next, 0.1 ml supernatant, 0.3 ml phosphate buffer, 0.3 ml methionine (750 mM), 0.3 ml nitro-blue tetrazolium (750 μM), 0.3 ml EDTA-Na_2_ (100 μM), 0.3 ml riboflavin (20 μM), and 0.5 ml distilled water were mixed by shaking. The mixed solution was exposed under 4000 lx illumination for 30 min. The maximum absorbance value was recorded using tubes that lacked the enzyme solution. The mixed solution without the enzyme solution under dark conditions served as the blank. The absorbance was recorded at 560 nm.

To determine the SI, seedlings were classified into five grades based on the degree of leaf injury. The grade standards are as follows: grade 0, no symptoms, no lesions on leaves; grade 1, a few leaf edges yellowing or withering; grade 2, about 50% of the leaf edges yellowing or withering; grade 3, most leaf edges withering or dead, and leaves prolapsed; grade 4, whole plant dead or dying. The calculation formula was SI = Σ (salt injury grade × number of plants at that grade)/total number of plants.

### Expression analysis

The following procedure was used for expression analysis. First, total RNA was extracted from various watermelon organs using the TRIzol method. Then reverse transcription was then performed using watermelon RNA as templates with the FastKing One Step RT-qPCR Kit (TIANGEN, China) to generate cDNA. Finally, qRT-RCR was measured using SYBR Green Master Mix (Vazyme, China). The primers are listed in Table S8.

### Subcellular localization assays

The full-length CDS of *ClWRKY61* was cloned into the pGreenII-GFP plasmid at the restriction sites (*Xho*I and *Eco*RV). The pGreenII-GFP-ClWRKY61 plasmids were transformed into *Agrobacterium.tumefaciens* GV3101 strain. The positive bacterial solution was shaken at 220 rpm and 28°C to an OD600 of 0.8–1.0. Subsequently, the turbid bacterial solution was centrifuged and the supernatant was discharged. The sediment was resuspended with MES buffer (10 mM 2-(*N*-morpholino) ethanesulfonic acid, 10 mM MgCl_2_, and 100 μM acetosyringone) to an OD600 of 0.4 and then injected into tobacco leaves. The lower epidermis of infected leaves was gently torn off and sectioned during 48–72 h. Fluorescence of tobacco cells was observed using a fluorescence microscope (Olympus, Japan). The primers are listed in Table S8.

### Plasmid construction for knockout and overexpression assays

Two specific sgRNA target sites for CRISPR/Cas9 were projected for the genes using the CRISPR-P 2.0 website (http://crispr.hzau.edu.cn/CRISPR2/). Primers containing sgRNA were used to amplify sgRNA-U6-26t-U6-29p-sgRNA cassettes using the pCBC-DT1T2 vector as a template. Then, it was introduced into the pBSE402 vector via the *Bsa*I restriction site. To obtain transgenic watermelon plants overexpressing *ClWRKY61*, the CDS of *ClWRKY61* was cloned into the pCAMBIA1305.4 vector at the restriction sites (*Bam*HI and *Pml*I). Green fluorescence protein (GFP) was expressed using the *35S* promoter, and positive plants were screened. The primers are listed in Table S8.

### Watermelon genetic transformation

After being disinfected with a 3% sodium hypochlorite solution, the seeds were placed on an agar medium and cultivated in the dark for 2 days. The cotyledons were infected with *Agrobacterium tumefaciens* strain EHA105 carrying CRISPR and overexpression vector. The genetic transformation material for *ClWRKY61* was ‘YL’ and that for *ClLEA55* was ‘TC’. Small pieces of cotyledon were cultured at 28°C in the dark for 72 h. The explants were cultured in MS medium containing 6-BA for approximately 15 days. GFP fluorescent-positive adventitious buds were selected using a fluorescent lamp (LUYOR-3415RG, USA).

### Yeast-two-hybrid assays

The CDS of *ClWRKY61* was inserted into the bait vector pGBKT7 via the *Bam*HI and *Eco*RI restriction sites. A 60 bp sequence was removed from the C-terminus to inhibit self-activation. Y2H assays were performed by mating the bait vector BD-ClWRKY61^D2^ with the entire cDNA library. The CDS of *ClLEA55* was inserted into the prey vector pGADT7 at the *Bam*HI and *Eco*RI restriction sites. pGBKT7-ClWRKY61^D2^ and pGADT7-ClLEA55 were co-transformed into the yeast Y2H strain and cultivated at 28°C in SD/-Trp-Leu/X-α-gal two deficiency selected media for 5–8 days. The blue monoclonal strain was then transferred to SD-Ade/-His/-Leu/-Trp/X-α-gal four deficiency selected media for 5–8 days at 28°C. The primers are listed in Table S8.

### Bimolecular fluorescence complementation assays

To generate BiFC vectors, the CDS of *ClWRKY61* was inserted into the nYFP vector pBI221NE resulting in ClWRKY61-nYFP. The CDS of *ClLEA55* was inserted into the cYFP vector pBI221CE resulting in ClLEA55-cYFP. Both vectors used *Xho*I and *Kpn*I restriction sites. Transient watermelon protoplast co-transformation of ClWRKY61-nYFP and ClLEA55-cYFP was conducted based on the previously expounded PEG-mediated transformation protocol [72]. YFP fluorescence of protoplasts was observed under a laser scanning confocal microscope (Leica, Germany). The primers are listed in Table S8.

### Pull-down assays

To generate the protein expression vectors, the CDS of *ClWRKY61^D2^* was inserted into the pET32a-His vector. The CDS of *ClLEA55* was inserted into the pGEX4T1-GST vector. The pET32a-ClWRKY61^D2^ and pGEX4T1-ClLEA55 plasmids were transformed into *E. coli* BL21 (DE3) strain and induced by 2 mM isopropyl beta-d-thiogalactopyranoside overnight at 18°C. Afterward, total bacterial proteins were extracted using ultrasound, and the His-ClWRKY61^D2^ proteins were purified using Ni-NTA agarose beads. The pull-down analysis was conducted through the Pierce™ GST Protein Interaction Pull-Down Kit (Thermo Fisher Scientific, USA). The GST-tagged proteins were immobilized using glutathione agarose. After purification, His-tagged fusion proteins were added to glutathione agarose with GST-tagged proteins and co-incubated for 2 h at 4°C. After elution, protein complexes were detected by SDS-PAGE and immunoblotting assays with antibodies against GST and His. The primers are listed in Table S8.

### Luciferase complementation imaging assays

To generate LCI vectors, the CDS of *ClWRKY61* was inserted into the pCAMBIA1300-nLUC vector and the CDS of *ClLEA55* was inserted into the pCAMBIA1300-cLUC vector. The restriction sites of both nLUC and cLUC vectors were *Kpn*I and *Sal*I. The constructed plasmids were introduced into the GV3101-psoup strain of *A. tumefaciens*. The positive bacterial solution was shaken to an OD600 of 0.8–1.0 at 220 rpm and 28°C. Subsequently, the turbid bacterial solution was centrifuged, and the supernatant was discharged. The sediment was resuspended with MES buffer to an OD600 of 0.4 and then co-injected into tobacco leaves. After 48–72 h, the backs of these leaves were evenly coated with fluorescein potassium salt solution and observed using a plant living imaging system (CCD, USA). The primers are listed in Table S8.

### RNA-seq assays

For transcriptome sequencing, the total RNA of leaf samples stored at −80°C was independently extracted from *crwrky61–1* and WT lines at 3 days after salt treatment and sent to Novogene Bioinformatics Technology Co., Ltd. (Beijing, China) for high-throughput sequencing. The double-stranded cDNA was synthesized for mRNA and the library was constructed by AMPure XP beads. Sequence using the Illumina NovaSeq6000 platform to obtain 150-bp paired-end reads. The clean data were mapped onto the watermelon reference genome (http://cucurbitgenomics.org/v2/) using HISAT2 v2.0.5. The DEGs were identified using DESeq2 software with the following standard, |log_2_-fold change| ≥ 1.0 and a false discovery rate ≤ 0.05.

### Statistical analysis

Statistical analysis of all data using IBM SPSS Statistics 19 software. Values were presented as the means ± standard deviation. The one-way ANOVA and Student’s *t*-test were used to confirm significant differences.

## Supplementary Material

Web_Material_uhae320

## Data Availability

The data that support the findings of this study are available in the Supplementary information of this article.
