# Supplementary material for: The transcription factor ClWRKY61 interacts with ClLEA55 to enhance salt tolerance in watermelon
Source: Hortic Res. 2024 Nov 11;12(3):uhae320. doi: 10.1093/hr/uhae320 (PMC11879168; doi:10.1093/hr/uhae320)
Supplement: Web_Material_uhae320 [file web_material_uhae320.zip › Figure - S.pdf]

A

MDCSWPDTTPSDRRKAADELLRGRELAQQLRAYLQRTSNSGGTASQDLLSRILTSFSKTLSILNRCDSDDINGSIVDSPEDRASRKSQESGDSCKSSDRRG
CYKRRKSCQSWARESCSLVD**DGHAWRKYGQKTILNAKYPRNYYRCTHKFDQACQATKQVQRLQDHPPKFRTTYYGHHTC**SNFLKASDIVLGSS
NFDDSCGVLLSFDTPAAPNFLLQQDAMLVKKEVAIAESRDDEAVCSPSDYISTAEPSPDDHLSEVFMGSVVDFEDDVLQFQF

B

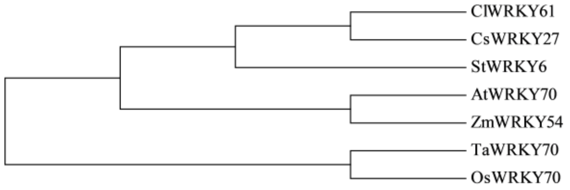

Fig. S1 Protein sequence of ClWRKY61 and phylogenetic analysis. A) The protein sequence of ClWRKY61. Yellow represents the conserved domain; red represents the WRKY motif; and green represents the zinc-finger motif. B) Phylogenetic analysis of ClWRKY61 and its closely related homologs from *Cucumis sativus* (CsWRKY27), *Solanum tuberosum* (StWRKY6), *Arabidopsis thaliana* (AtWRKY70), *Zea mays* (ZmWRKY54), *Triticum aestivum* (TaWRKY70), and *Oryza sativa* (OsWRKY70).

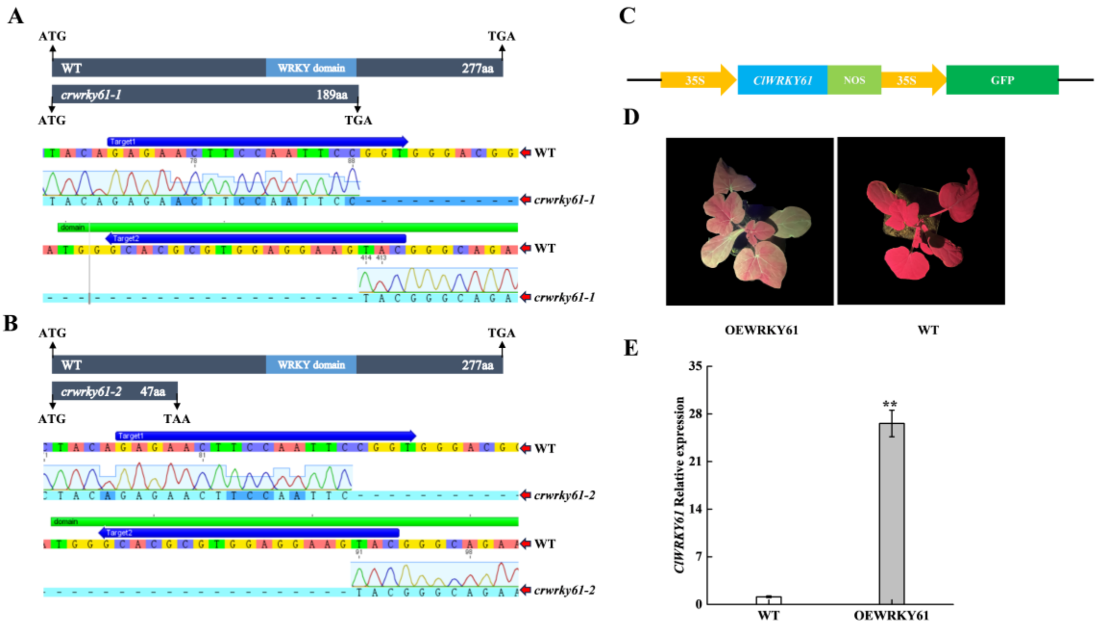

Fig. S2 Genotypic identification of *ClWRKY61* knockout and overexpression lines. A) Sanger sequencing chromatogram data of the transgenic homozygous mutant *crwrky61-1*.A total of 88 amino acids were deleted between the two target sites. B) Sanger sequencing chromatogram data of the transgenic homozygous mutant *crwrky61-2*. A premature stop codon generated a truncated protein of 47 amino acids.

C) Construction of the *ClWRKY61* overexpression vector. D) Fluorescence of overexpression and wild-type seedlings. E) Expression analysis of *ClWRKY61 in* overexpression lines. Data were presented as mean ± SD (n = 3). Asterisks indicate statistically significant differences (**P < 0.01).

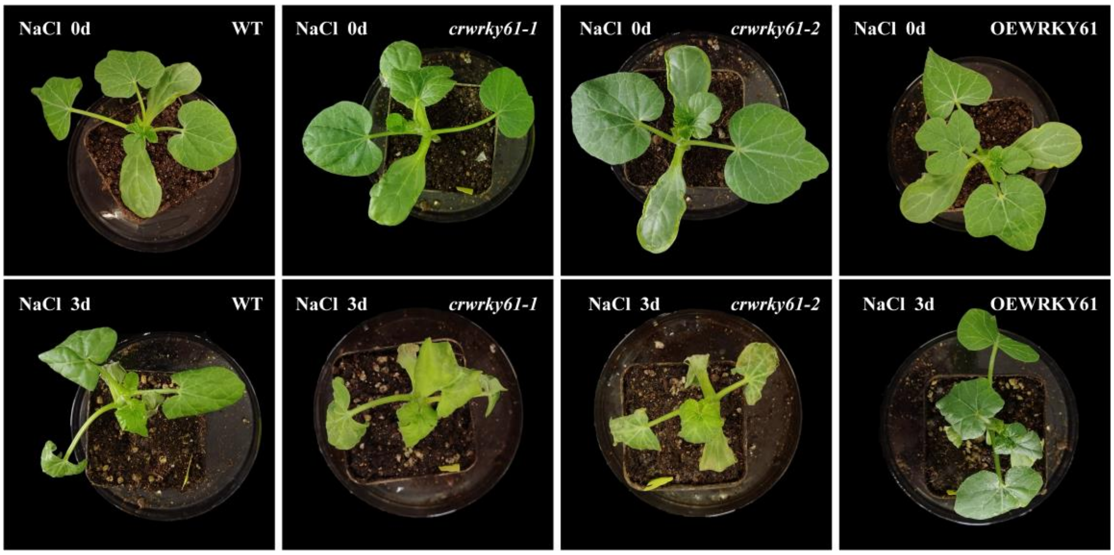

Fig. S3 Phenotypic comparison of transgenic plants of *ClWRKY61* under salt stress. Phenotypic comparison of knockout, overexpression, and wild-type seedlings of *ClWRKY61* grown under 300 mM NaCl treatment for 3 days.

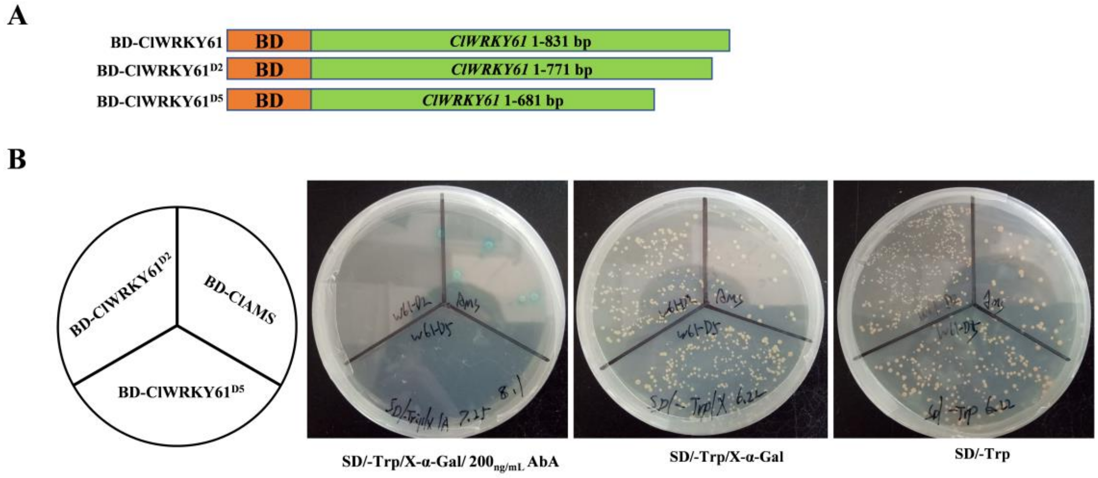

Fig. S4 Self-activation inhibition of *ClWRKY61* in yeast-two-hybrid (Y2H) assays. A) Construction of the pGBKT7 (BD) vector for *ClWRKY61*. *ClWRKY61*^{D2} is *ClWRKY61* with, 60bp of the base from the C-terminus removed. *ClWRKY61*^{D5} is *ClWRKY61* with, 150bp of the base from the C-terminus removed. B) *ClWRKY61*^{D2} and *ClWRKY61*^{D5} do not have blue spots grown on SD/-Trp/X-α-Gal/ 200$_{ng/mL}$ AbA. ClAMS served as the

control.

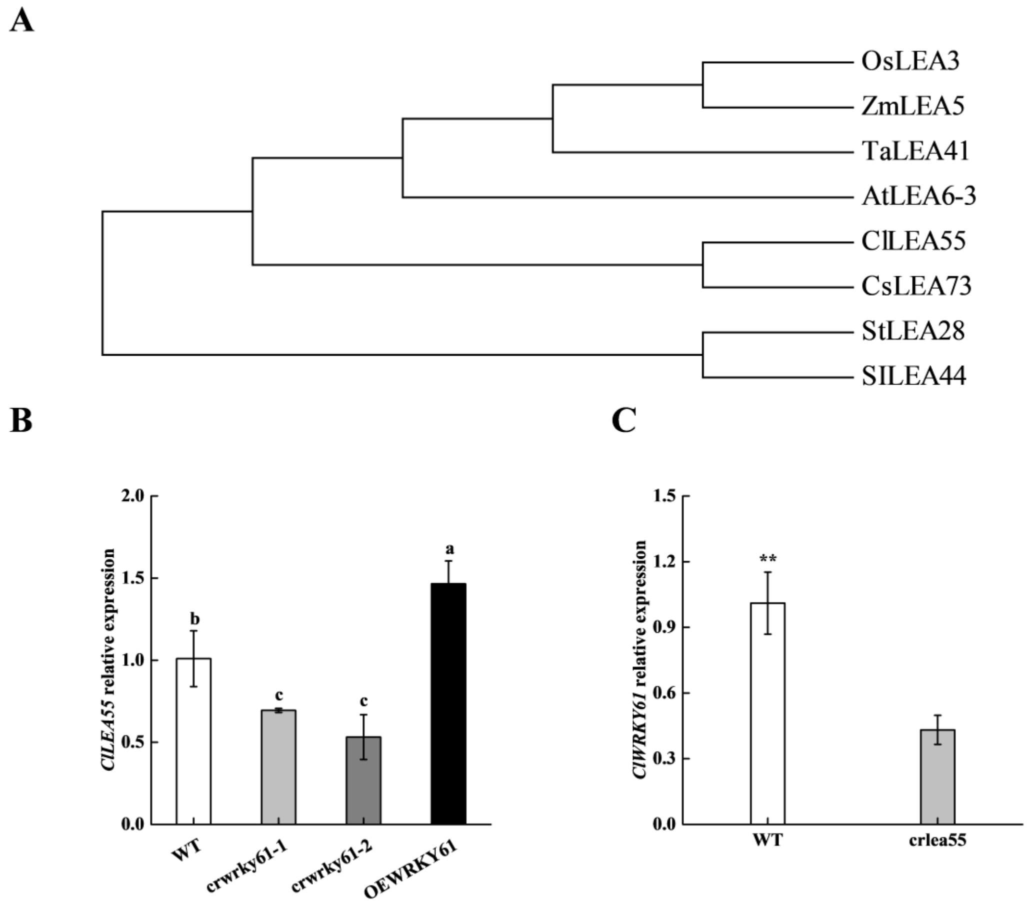

Fig. S5 Phylogenetic analysis of ClLEA55 and expression analysis of *ClLEA55* and *ClWRKY61* in transgenic plants. A) Phylogenetic analysis of ClLEA55 and its closely related homologs from *Oryza sativa* (OsLEA3), *Zea mays* (ZmLEA5), *Triticum aestivum* (TaLEA41), *Arabidopsis thaliana* (AtLEA6-3), *Cucumis sativus* (CsLEA73), *Solanum tuberosum* (StLEA28), and *Solanum lycopersicum* (SlLEA44). B) Expression analysis of *ClLEA55* in wild-type, knockout, and overexpression lines of *ClWRKY61*. C) Expression analysis of *ClWRKY61* in wild-type and knockout lines of *ClLEA55*. Data are presented as mean ± SD (n = 3). Different lowercase letters and asterisks indicate statistically significant differences (one-way ANOVA, $^{**}P < 0.01$).

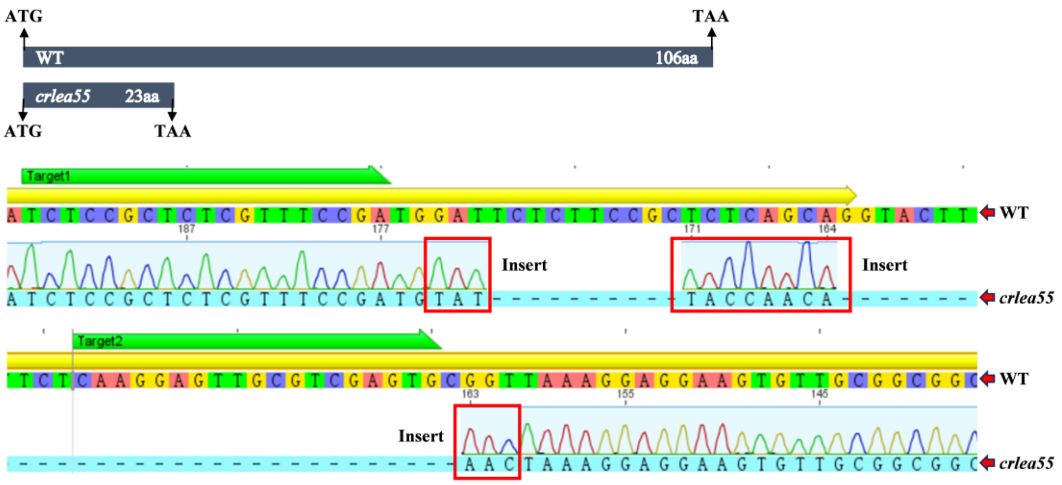

Fig. S6 Genotypic identification of *ClLEA55* knockout lines. Sanger sequencing chromatogram data of the transgenic homozygous mutant *crlea55*. A premature stop codon generated a truncated protein of 23 amino acids, along with a 73bp deletion and 14bp insert.

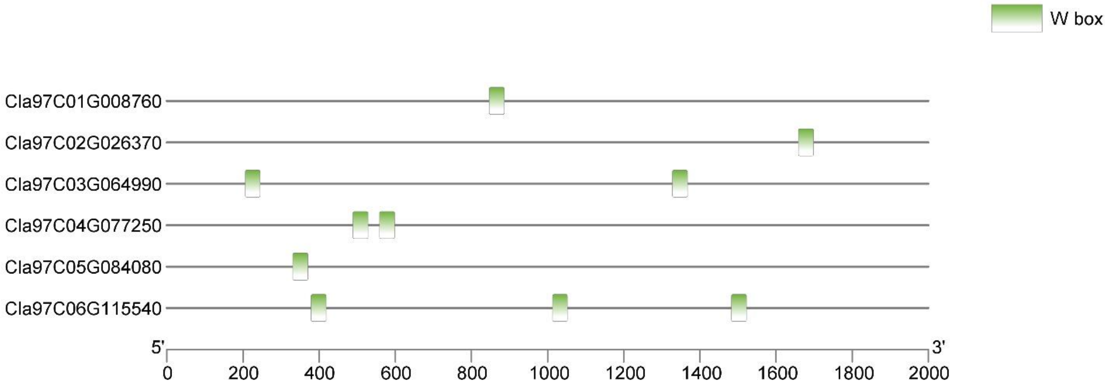

Fig. S7 W-box site analysis in up-regulated DEGs in the *crwrky61-1* vs. wild-type comparison group under salt stress.

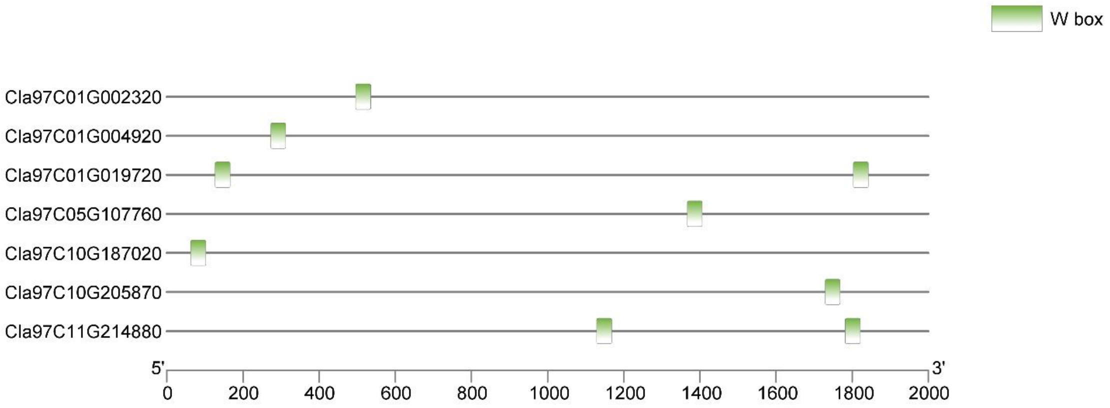

Fig. S8 W-box site analysis in down-regulated DEGs in the *crwrky61-1* vs. wild-type comparison group under salt stress.
