# Supplementary material for: The transcription factor ClWRKY61 interacts with ClLEA55 to enhance salt tolerance in watermelon
Source: Hortic Res. 2024 Nov 11;12(3):uhae320. doi: 10.1093/hr/uhae320 (PMC11879168; doi:10.1093/hr/uhae320)
Supplement: Web_Material_uhae320 [file web_material_uhae320.zip › Table S6 and S7.docx]

Table S6 The information of up-regulated genes in the wild-type vs. *crwrky61-1* line comparison group by RNA-Seq and qRT-PCR analysis under salt stress.

| Gene ID | Ara_ID | Functional annotation |
| --- | --- | --- |
| Cla97C01G008760 | AT5G17230 | Encodes phytoene synthase that is the rate-limiting enzyme in the carotenoid biosynthetic pathway and that interacts with the ORANGE (OR) protein. |
| Cla97C02G026370 | AT4G38620 | Encodes MYB4 DNA-binding protein |
| Cla97C03G064990 | AT3G43190 | Encodes a protein with sucrose synthase activity |
| Cla97C04G077250 | AT1G73480 | alpha/beta-Hydrolases superfamily protein |
| Cla97C05G084080 | AT3G24170 | Encodes a cytosolic glutathione reductase |
| Cla97C06G115540 | AT5G26340 | Encodes a protein with high affinity, hexose-specific/H^+^ symporter activity. |

Table S7 The information of down-regulated genes in the wild-type vs. *crwrky61-1* line comparison group by RNA-Seq and qRT-PCR analysis under salt stress.

| Gene ID | Ara_ID | Functional annotation |
| --- | --- | --- |
| Cla97C01G002320 | AT1G64065 | Late embryogenesis abundant (LEA) hydroxyproline-rich glycoprotein family |
| Cla97C01G004920 | AT5G51190 | encodes a member of the ERF (ethylene response factor) subfamily B-3 of ERF/AP2 transcription factor family. |
| Cla97C01G019720 | AT5G66210 | Calcium dependent protein kinase |
| Cla97C05G107760 | AT1G10760 | Encodes an α-glucan, water dikinase required for starch degradation |
| Cla97C10G187020 | AT5G47230 | Encodes a member of the ERF (ethylene response factor) subfamily B-3 of ERF/AP2 transcription factor family. |
| Cla97C10G205870 | AT2G38470 | Member of the plant WRKY transcription factor family. |
| Cla97C11G214880 | AT1G71520 | Encodes a member of the DREB subfamily A-5 of ERF/AP2 transcription factor family |
